# Effects and feasibility of hyperthermic baths in comparison to exercise as add-on treatment to usual care in depression: a randomised, controlled pilot study

**DOI:** 10.1186/s12888-020-02941-1

**Published:** 2020-11-11

**Authors:** Johannes Naumann, Iris Kruza, Luisa Denkel, Gunver Kienle, Roman Huber

**Affiliations:** 1 European Institute for Physical Therapy and Balneology, Freiburg, Germany; 2 University of Freiburg, Faculty of Medicine, Freiburg, Germany

## Abstract

**Background:**

Limitations of current therapy of depression highlight the need for an immediately available, easily implementable add-on treatment option with high acceptance from patients. Hyperthermic baths (HTB) are a form of balneotherapy with head-out-of-water-immersion in a hot pool or tub at 40 °C for 15–20 min. A prior study suggests that HTB added to usual depression care can have antidepressant effects.

**Method:**

Single-site, open-label randomised controlled 8-week parallel-group pilot study at a university outpatient clinic. 45 medically stable outpatients with moderate depression as determined by the 17-item Hamilton Depression Rating Scale (HAM-D) score ≥ 18 and a score ≥ 2 on item 1 (Depressed Mood) were recruited. They were randomised to twice weekly HTB (*n* = 22) or a physical exercise program (PEP) of moderate intensity (*n* = 23). Primary outcome measure was the change in HAM-D total score from baseline (T0) to the 2-week time point (T1). Linear regression analyses, adjusted for baseline values, were performed to estimate intervention effects on an intention-to-treat (ITT) and per-protocol (PP) principle.

**Results:**

Forty-five patients (HTB *n* = 22; PEP *n* = 23) were analyzed according to ITT (mean age = 48.4 years, SD = 11.3, mean HAM-D score = 21.7, SD = 3.2). Baseline-adjusted mean difference after 2 weeks was 4.3 points in the HAM-D score in favor of HTB (*p* < 0.001). Compliance with the intervention and follow-up was far better in the HTB group (2 vs 13 dropouts). Per protocol analysis only showed superiority of HTB as a trend (*p* = 0.068). There were no treatment-related serious adverse events. Main limitation: the number of dropouts in the PEP group (13 of 23) was higher than in other trials investigating exercise in depression. Due to the high number of dropouts the effect in the ITT-analysis may be overestimated.

**Conclusions:**

HTB added to usual care may be a fast-acting, safe and easy accessible method leading to clinically relevant improvement in depression severity after 2 weeks; it is also suitable for persons who have problems performing exercise training.

**Trial registration:**

German Clinical Trials Register (DRKS) with the registration number DRKS00011013 (registration date 2016-09-19) before onset of the study.

## Background

Depression contributes to significant economic burden and is associated with comorbid diseases (i.e. cardiovascular disease), and impaired health-related quality of life and functioning [1–5]. Despite advances in the treatment of depression, one-third of depressed patients fail to respond to conventional antidepressant medication [6]. Moreover, current medications cause significant side effects in the central-nervous system and commonly used antidepressants have a delayed onset of action, further highlighting the need for faster acting, easy available and more effective treatments with fewer side effects [7–9].

### Hyperthermia - an ancient treatment for mental illness

Fever, respectively hyperthermia, has been used as a medical treatment and part of balneotherapy since ancient times, and beneficial effects of fever on mental illness were already described in antiquity [10, 11]. Evidence suggests that hyperthermic baths (HTB) performed in a pool or tub with a water temperature of 40 °C and other forms of whole-body hyperthermia (WBH) have anti-depressant effects, mediated through changes in circadian rhythm, temperature physiology and sleep, which are disturbed in depressive patients [12–14]. After 15–20 min HTB a raise in core body temperature of 1.7 °C can be expected [15, 16]. Body core temperature in depressed patients is elevated during the night, while sleep quality is best when the core body temperature decreases; thus, change of body temperature might improve sleep quality. Findings from experimental studies show that manipulation of core and skin temperatures can improve or disrupt sleep, and it is well-known that sleep disruption negatively influences quality of life, enhances length and severity of episodes and contributes to relapse in depression [13, 17, 18]. Strengthening sleep promoting brain activity improves depression (e.g. by sleep deprivation and anti-depressant medication) [19, 20]. In addition, a novel hypothesis describes that an evolutionarily ancient thermo-afferent pathway, signaling from serotonergic sensory cells in the skin (Merkel cells) to serotonergic neurons and depression-related circuits in the brain, might explain the antidepressant effects of HTB [7].

### HTB - evidence

There is growing scientific evidence that HTB and WBH might be efficacious for treatment of depressive disorders [15, 21–23]. The results of a non-controlled HTB study with 20 depressive patients showed an improvement in the 21-item Hamilton Depression Rating Scale [24] after five baths [21]. HTB (especially before bedtime) improved sleep in healthy subjects [25–28], insomniac people [29, 30] and elderly patients with vascular dementia [31]. In a further non-controlled study using a radiant system to induce WBH, a single session showed a significant reduction in the Centers for Epidemiologic Studies Depression Scale [32] in 16 depressive patients [23]. In a randomised, sham-controlled study from the same group this favorable result of a single WBH session could be corroborated [22]. Our results of a randomised placebo-controlled pilot study using HTB compared with a sham light treatment as Placebo were also promising; after four interventions (2 weeks), the intention-to-treat analysis showed a significant (*p* = 0.037) difference in the 17-item Hamilton Depression Rating Scale (HAM-D) total score of 3.14 points in favor of the HTB group [15]. Regarding the superiority of HTB to a Placebo we now decided to compare HTB with physical exercise as an active control.

### Physical exercise in depression

Several meta-analyses [33–35], including three Cochrane reviews [36–38], have supported the antidepressant effects of exercise. Various psychobiological mechanisms are understood to play a role [34]. Both, aerobic exercise (e.g. running, cycling) and anaerobic exercise (e.g. strength-training) are effective in the treatment of depression. According to national and international guidelines for depression [39–41] physical activity is recommended as add-on therapy to pharmacotherapy.

After we found HTB to be superior to a Placebo (sham light therapy, [15]), our primary objective was to compare the efficacy of HTB with a proven non-pharmacological standard intervention - a physical exercise program (PEP). Secondary objectives were to evaluate safety (incidence of treatment discontinuation and adverse events (AE)) and feasibility of HTB applied in a home-setting.

## Methods

### Study design

Eight-week single-site, parallel-group, open-label randomised controlled pilot trial of HTB vs PEP for patients with a diagnosis of depression according to ICD-10 (F32/F33) of at least 4 weeks duration. Patients were recruited from the Medical Center - University of Freiburg. The study was registered in the German Clinical Trials Register (DRKS) with the registration number DRKS00011013 (https://www.drks.de/drks_web/; registration date 2016-09-19) before onset of the study. The study was conducted in accordance with the Declaration of Helsinki and local laws and regulations. All the participants filled in a written informed consent form before entering the study.

### Eligibility criteria

Eligible participants were to meet the following criteria: (1) Medically stable outpatients with a diagnosis of depressive disorder (ICD-10: F32/F33; criteria for single or recurrent depression without psychotic features) confirmed by a physician or psychotherapist; (2) men and women between18 and 65 years of age; (3) a moderate level of depressive symptoms assessed with the 17-item Hamilton Depression Rating Scale (HAM-D) total score ≥ 18 and a score ≥ 2 on item 1 (Depressed Mood) at screening and at baseline; (4) on a consistent antidepressant regimen or off antidepressant therapy for at least 4 weeks prior to baseline; (5) no changes in antidepressant treatment to be expected during the study.

Exclusion criteria included the presence of severe concomitant disease (i.e. heart failure New York Heart Association (NYHA) III-IV, cancer, tuberculosis, liver cirrhosis), contraindication for hot baths (i.e. open wounds, heat urticaria, varicosis, multiple sclerosis, epilepsy, orthostatic dysregulation), organic psychotic disorders, schizophrenia, hallucinations, bipolar disorders, dissociative personality disorder, suicidal thoughts, abuse of alcohol or other drugs within the last 6 months, use of ß-blockers or corticosteroids, pregnancy, lactation, and participation in clinical trials in the 8 weeks preceding the study. We excluded patients over age 65 because HTB causes circulatory stress and the risk of cardiovascular disease increases with age [41, 42] and because symptoms of depression may be expressed differently in late-life depression [43].

### Interventions

Patients were randomly assigned to receive either HTB or PEP for 8 weeks with two interventions per week (Table 1). Patients were told that two promising treatments were being compared in the study. The attention and time spent with the patients was similar in both groups.

**Table 1:** Trial design

	Screening	Baseline/Randomisation (T0)	After 4 interventions (T1)			After 16 interventions (T2)
		0 wk	2 wk	4 wk	6 wk	8 wk
HTB/PEP target (n)^a^			4	8	12	16
HAM-D^b^	x	x	x			x
Medical history		x				
BDI-II^c^		x	x			x
PSQI^d^		x	x			x
Global judgement of efficacy			x			x
Global judgement of tolerability			x			x
Change of medication/therapy			x			x
Adverse events^e^			x	x	x	x
Telephone contact				x	x	

^a^ HTB: hyperthermic baths, PEP: physical exercise program

^b^ HAM-D: 17-item Hamilton Depression Rating Scale

^c^ BDI-II: Beck Depression Inventar II

^d^ PSQI: The Pittsburgh Sleep Quality Index

^e^ Adverse events were documented before and after each treatment

### Hyperthermic baths (HTB)

HTB were applied as head-out-of-water-immersion in a 40 °C pool at a thermal bath near Freiburg, Germany. All the baths were taken in the afternoon (14:00–18:00). Five patients could sit in the pool at a time. The baths were taken until the patients noticed discomfort, the target being 20 min [16]. Directly after the bath, the patients were accompanied to a nearby resting room, where they lay down and wrapped in warm blankets with two conventional 0.7 l hot water bottles (abdomen, thighs) filled with hot water (ca. 70 °C) for at least another 20 min to keep the body temperature elevated. After 20 min in a pool with a water temperature of 40 °C, a raise in core body temperature of 1.7 °C is to be expected [16]. After four sessions under supervision (IK, LD), the remaining 12 sessions were performed by the patients themselves at the thermal bath or in the home-setting. At home, the baths were performed in the same way (water temperature 40 °C, duration 20 min). The supervisors documented the exact duration of the bath and the body temperature. In case that a patient felt uncomfortable the bath was terminated. After each bath patients were asked if there were any AE. All AE reported spontaneously by patients or observed by the supervisors were recorded in prepared data sheets. After the fourth application the patients were instructed in written and oral form to perform the following baths in the thermal bath or at home on their own as before and to document temperature, duration and AE in a prepared diary (S1 File).

### Physical exercise program (PEP)

Patients in the PEP group took part in a structured program of moderate intensity which mainly followed the recommendation in international guidelines [39, 40]. It consisted of warming-up, walking, jogging, stretching and strengthening elements for about 45–50 min, conducted outside in small groups of five patients each. The first four sessions took place under supervision (IK, LD; trained in exercise therapy). The following 12 sessions were performed by the patients themselves in groups or alone. They received a detailed explanation and written instructions (S2 File) and were asked to carry out the guided programme as they learned it in the supervised sessions. Training duration and the exercise components completed were documented in prepared diaries. Patients of both groups received a telephone call from the study personal 4 and 6 weeks after randomisation to document and reinforce compliance.

### Outcomes

The primary outcome was observer-rated and interview-based severity of depression assessed by the 17-item HAM-D at 2 weeks (T1), determined by the change in HAM-D total score at T1 relative to T0 (baseline). The HAM-D has a score range of 0 (least depressed) to 52 (most depressed), with good reliability and validity [44].

Further prespecified clinical secondary outcomes were: Patients’ subjective perception of depression severity as measured by the self-report Beck Depression Inventory II (BDI-II) [45, 46]. The BDI-II consists of 21 items, each rated 0–3 according to severity of difficulties experienced, with a possible range of 0–63 (minimal 0–13, mild 14–19, moderate 20–28, or severe ≥29). Sleep quality was assessed with the Pittsburgh Sleep Quality Index (PSQI) [47–49]. The PSQI is a self-rating questionnaire resulting in a global score between 0 and 21, with higher scores indicating poorer sleep quality. A global score > 5 is an indicator of relevant sleep disturbances. All mentioned questionnaires were used in the validated German version [50].

Unblinded assessments (IK, LD received prior training and were supervised by RH) were performed at the following three time points (Table 1): before start of HTB treatment (T0), immediately on completion of the two-week treatment interval (T1), according to results that effects are supposed to appear early [12] and at the end of treatment (T2).

### Core body temperature

Core body temperature was measured with an infrared-ear-thermometer (Thermoscan®, Type: 6021, Braun GmbH). In the HTB group, the core body temperature was measured directly before and after the bath and after resting; in the PEP group directly before and after the exercise sessions.

### Global judgment of efficacy and tolerability

After four treatments (T1) and after end of treatment (T2), patients were asked to rate the efficacy and tolerability of the intervention on a 5-point scale (1 = very good; 2 = good; 3 = moderate; 4 = absent; 5 = worsening).

### Safety and feasibility outcomes

To estimate the safety of HTB, the incidence of treatment discontinuation and the occurrence of AE were evaluated. To answer the feasibility question, information regarding compliance and experiences of the patients were evaluated. In order to improve adherence to intervention procedures contact was made by phone after four and 6 weeks, respectively.

### Adverse events

All AE reported spontaneously by patients or observed by the assistants were recorded before and after each treatment. If a serious adverse event (SAE) occurred, the principal investigator took all the necessary and appropriate measures to ensure the safety of the patient.

### Sample size estimation

No data on effect size of HTB in comparison to PEP were available; however, from the results of our previous study [15], we expected a moderate to large effect size for HTB relative to control [33]. A between-group effect size of *d* = 0.91 and 1-β = 0.80 power was chosen for this pilot study which required 40 patients (*n* = 20 per arm) to reach significance with α = 0.05. This estimation was based on the primary outcome of depression symptoms as measured by the HAM-D total score at 2 weeks of intervention [51]. However, this sample size does not account for subgroup analyses.

### Randomisation and blinding

All eligible patients who consented to participate were allocated to HTB or PEP after baseline assessment in a 1:1 ratio by simple randomisation without blocking or stratification. Randomisation codes were computer-generated by an independent biometric center. Allocation was performed with opaque sealed envelopes that were randomly chosen by the participants. Enrolment (RH) was done directly after informed consent was obtained from the patients. Both therapies could not be blinded. Outcome assessment (IK, LD) was unblinded. Data management and analyses were performed blinded to treatment allocation.

### Statistical analyses

Treatment comparison of the primary endpoint (HAM-D change from baseline to T1) was performed within a generalized linear regression model adjusted for baseline values. BDI-II and PSQI were analyzed in the same way. Analyses were done on the intention-to-treat (ITT) population, defined as all allocated patients, applying the last-observation-carried-forward (LOCF) approach to impute missing data. Baseline characteristics were compared using 2-sided *t* tests for continuous data and χ^2^ statistics. The per-protocol (PP) population was defined as all patients who had a complete dataset for the primary outcome and had participated in at least 75% of the treatments, meaning at least 3 of 4 treatments for T1, and at least 12 of 16 treatments for T2. We report *p*-values with the significance level set at *p* < 0.05. The study followed a protocol (S1-S3 Protocol) and reporting followed the CONSORT (Consolidated Standards of Reporting Trials; Statement extension for nonpharmacological treatments; S4 Protocol). Prespecified secondary analyses were not adjusted for multiple comparisons and should therefore be regarded as descriptive and exploratory. Statistical analyses were performed using IBM® SPSS®, Version 24, for Windows.

### Data collection and monitoring

A data manager (CS), blind to treatment allocation, reviewed and evaluated data, in order to detect errors during data collection, and conducted a quality review of the database, with double data entry by two independent persons for 20% of the values.

## Results

### Enrolment and sample description

**Fig. 1:**
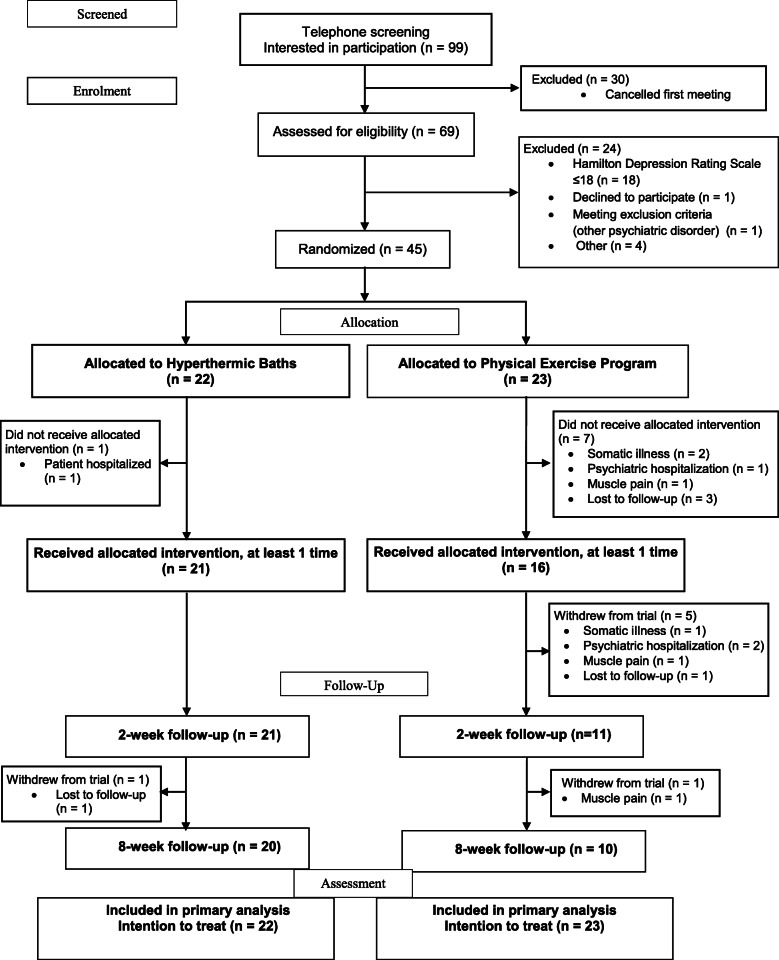
CONSORT flow diagram of study participants

The HTB study began recruiting patients in September 2016 and closed recruitment in January 2017. Sixty-nine adults agreed to participate and were assessed for eligibility. Of that number, 45 underwent randomisation (Fig. 1).

Randomisation was balanced with respect to demographic and clinical characteristics with more females (*p* = 0.047), a higher BMI (*p* = 0.135) and longer duration of depressive disorder in the PEP-group (*p* = 0.035), (Table 2). The mean depression severity score based on the HAM-D was 21 (HTB), respectively 22 (PEP), consistent with moderate severity depression [44]. Self-reported severity and symptoms of depression assessed by the BDI-II was 29 (HTB), respectively 31 (PEP), consistent with the lowest possible score for severe depression. Most patients had depression for several years; the shortest duration of depression was 2 months, the longest more than 20 years. Sleep quality, as measured by the PSQI total score (ranging from 0 to 21; score ≤ 5 associated with good sleep quality; score > 5 associated with poor sleep quality), was poor with a total score of 10 (HTB), respectively 11 (PEP). Prescription rates of antidepressants were high at baseline (HTB, 68%; PEP, 48%), as well as for psychotherapy (HTB, 71%; PEP, 83%).

**Table 2:** Demographic and clinical characteristics at baseline

	Overall (***N*** = 45)	Hyperthermic Baths (***N*** = 22)	Physical Exercise Program (***N*** = 23)	p
Gender: Female, *n* (%)	33 (73.33%)	13 (59.09%)	20 (86.96%)	0.047^c^
Age (years)	48.4 (11.3)	45.6 (12.3)	51.0 (9.8)	0.106
BMI (kg/m^2^)	25 (5)	24 (4.2)^b^	26 (5.6)^§^	0.135
Median (IQR)	24.5 (7)	23.5 (5)	26 (11)	
Duration of depression, years	6 (7)	4 (6)	8 (7.5)	0.035
Psychiatric hospital stays (*n*) (last 2 years)	21 (47.72%)	11 (52.38%)^a^	10 (43.47%)	0.763
Use of psychopharmaca, *n* (%)	26 (57.77%)	15 (68.18%)	11 (47.82%)	0.231
Psychotherapy	34 (77.27%)	15 (71.42%)^a^	19 (82.61%)	0.481
Sport, *n* (%)	32 (74.42%)	15 (75.0%)^b^	17 (73.91%)	1.0^c^
Baths, *n* (%)	14 (32.56%)	7 (35.0%)^b^	7 (30.43%)	1.0
HAM-D total score	21.7 (3.2)	21.2 (3.3)	22.2 (3.1)	0.322
Median (IQR)	21 (5)	21 (18–29)	22 (18–29)	
BDI-II	30.0 (7.1)	29.0 (5.8)	31.1 (8.1)	0.318
Median (IQR)	31 (8)	30.5 (19–38)	31 (13–50)	
Median (IQR)	10.8 (3.7) 10 (6)	10.2 (3.8) 10 (4–16)	11.4 (3.7) 11 (5–18)	0.300

Where not otherwise indicated, data are shown as mean and standard deviation (SD).

*IQR* interquartile range

*BMI* Body Mass Index, *HAM-D* 17-item Hamilton Depression Rating Scale, *BDI-II* Beck Depression Inventar II, *PSQI* The Pittsburgh Sleep Quality Index

^a^missing *n* = 1; ^b^missing *n* = 2;

^c^chi-quadrat-test, Fisher’s exact test

Primary outcome data (HAM-D total score after 2 weeks) were available for 32 patients, representing a loss to follow-up of 29% (5% in the HTB group and 52% in the PEP group). Eight patients (HTB *n* = 1; PEP *n* = 7) discontinued treatment without receiving their allocated intervention. A further five patients (PEP group) withdrew from trial before T1; another two after T1 (HTB n = 1; PEP n = 1).

### Treatment effect on core body temperature

Core body temperature rose from 36.7 °C before the bath to 38.6 °C directly after the bath (mean change 1.96 °C, standard deviation [SD] 0.5), and maintained at 37.4 °C (mean change 0.7 °C, SD = 0.5) after rest. The mean temperature of the bath was 40.2 °C (SD = 0.3). Mean duration of baths was 20.26 min (SD = 1.74) and resting time amounted to 21.7 min (SD = 2.87). In the PEP group, there was no difference in core body temperature before or after physical exercise (36.48 °C, SD = 0.46, respectively 36.50 °C, SD = 0.52). Mean duration of exercises was 56.44 min (SD = 17.55).

### Primary outcome

The ITT analysis showed an adjusted mean difference between the groups of 4.3 points (95% CI 2.16 to 6.42; ß = 0.438) in the HAM-D score after 2 weeks in favor of HTB (*p* < 0.001, Table 3) using LOCF for missing data. The PP analyses showed an adjusted mean difference between the groups of 2.7 points (95% CI − 0.22 to 5.69) in the HAM-D score after 2 weeks in favor of HTB (*p* = 0.07, Table 4). Further analysis using different methods (linear regression) for substituting missing data also showed a non-significant difference in favor of the HTB group.

**Table 3:** Between-group differences in the intent-to-treat last observation carried forward sample

Outcome Measure	HTB Group (***N*** = 22)	PEP Group (***N*** = 23)	HTB vs PEP Adjusted mean difference^**c**^ (95% CI)	p^**c**^
HAM-D total score				
Baseline	21.2 (3.3)	22.2 (3.1)		
T1: 2 weeks^a^	15.8 (4.4)	20.8 (4.2)	4.3 (2.16 to 6.42)	**< 0.001**
T2: 8 weeks^b^	15.3 (5.8)	19.0 (6.1)	2.9 (−0.40 to 6.14)	0.084
BDI-II				
Baseline	29.0 (5.8)	31.1 (8.1)		
T1: 2 weeks^b^	20.3 (8)	29.1 (8.8)	7.5 (2.99 to 11.93)	**0.002**
T2: 8 weeks^b^	20.5 (10.8)	24.8 (11.8)	3.1 (−3.36 to 9.53)	0.340
PSQI				
Baseline	10.2 (3.8)	11.4 (3.7)		
T1: 2 weeks^b^	8.7 (3.7)	11.5 (4.1)	2.0 (0.14 to 3.92)	**0.036**
T2: 8 weeks^b^	8.3 (3.2)	10.7 (4.1)	1.8 (−0.13 to 3.64)	0.067

*Abbreviations: HAM-D* 17-Item Hamilton Depression Rating Scale, *BDI-II* Becks Depression Inventar II, *PSQI* The Pittsburgh Sleep Quality Index

Data are shown as mean and standard deviation (SD).

^a^Primary outcome

^b^Prespecified secondary outcome

^c^Linear regression analyses adjusted for baseline scores

**Table 4:** Between-group differences in the per-protocol completer sample

Outcome Measure	HTB Group	PEP Group	HTB vs PEP Adjusted mean difference^**§**^ (95% CI)	p^**c**^
HAM-D total score				
Baseline	21.0 (3.2)	21.4 (3.0)		
T1: 2 weeks^a^	14.9 (3.6)	17.9 (4.2)	2.7 (−0.22 to 5.69)	0.07
T2: 8 weeks^b^	14.1 (5.2)	12.9 (5.1)	−0.9 (−5.66 to 3.97)	0.72
BDI-II				
Baseline	28.5 (6.1)	32.0 (4.1)		
T1: 2 weeks^a^	19.2 (7.8)	24.2 (6.9)	3.0 (−3.09 to 9.08)	0.32
T2: 8 weeks^b^	18.0 (10.7)	13.0 (6.5)	−7.7 (−16.19 to 0.76)	0.07
PSQI				
Baseline	9.9 (3.9)	10.6 (3.0)		
T1: 2 weeks^a^	8.3 (3.8)	9.6 (3.9)	0.9 (−1.83 to 3.57)	0.51
T2: 8 weeks^b^	8.1 (3.6)	8.0 (3.9)	−1.2 (−3.74 to 1.39)	0.35

Data are shown as mean and standard deviation (SD).

*Abbreviations: HAM-D* 17-Item Hamilton Depression Rating Scale, *BDI-II* Becks Depression Inventar II, *PSQI* The Pittsburgh Sleep Quality Index

^a^ HTB *n* = 19; PEP *n* = 9; ^b^ HTB *n* = 15; PEP *n* = 7

^c^Linear regression analyses adjusted for baseline scores

### Descriptive secondary analyses

**Fig. 2:**
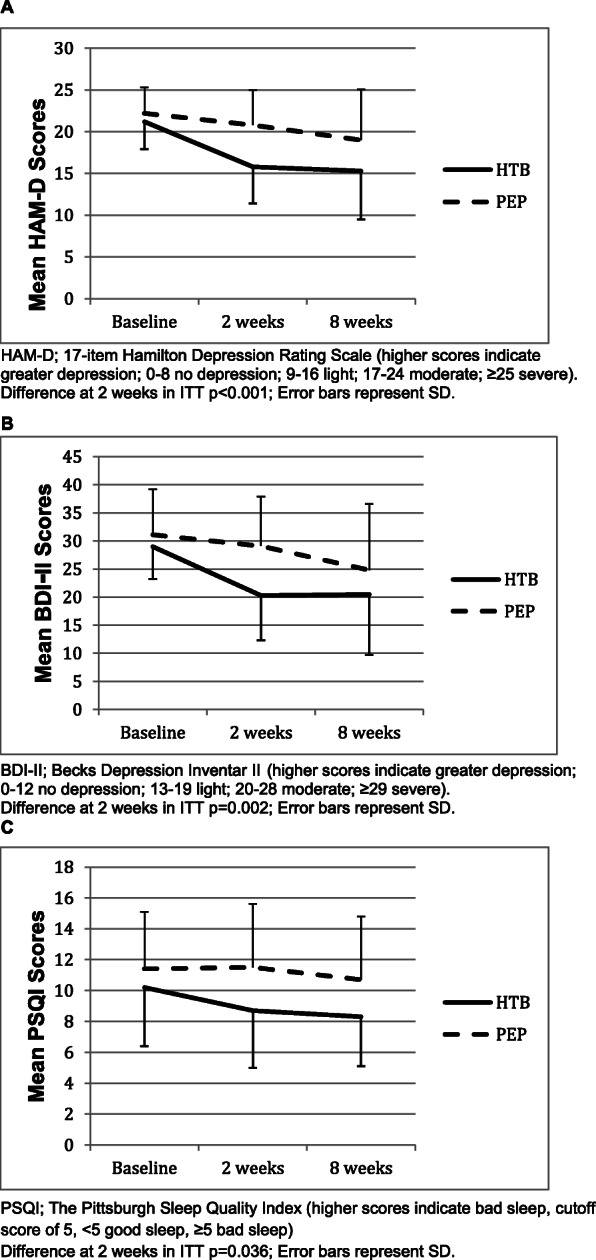
Modeled change scores by week. **a** HAM-D; 17-item Hamilton Depression Rating Scale (higher scores indicate greater depression; 0–8 no depression; 9–16 light; 17–24 moderate; ≥25 severe). Difference at 2 weeks in ITT *p* < 0.001; Error bars represent SD. **b** BDI-II; Becks Depression Inventar II (higher scores indicate greater depression; 0–12 no depression; 13–19 light; 20–28 moderate; ≥29 severe). Difference at 2 weeks in ITT *p* = 0.002; Error bars represent SD. **c** PSQI; The Pittsburgh Sleep Quality Index (higher scores indicate bad sleep, cutoff score of 5, < 5 good sleep, ≥5 bad sleep). Difference at 2 weeks in ITT *p* = 0.036; Error bars represent SD

Analysis of secondary outcomes was exploratory and no adjustments for multiple testing were applied. Looking at the mean differences compared to baseline, the HAM-D results show a stable improvement in the HTB group of 5.4 points after 2 weeks and of 5.9 points after 8 weeks in the ITT-analysis and an improvement in the HTB group of 6.1 points after 2 weeks and of 6.9 points after 8 weeks in the PP-analysis, whereas in the PEP group we see an improvement of 1.4 points after 2 weeks and of 3.2 points after 8 weeks in the ITT-analysis (Fig. 2a) and an improvement of 3.5 points after 2 weeks and of 8.5 points after 8 weeks in the PP-analysis.

For the following results only the ITT-analysis is reported. A similar pattern can be seen in the results of the BDI-II, with a mean difference compared to baseline in the HTB group of 8.7 points after 2 weeks and of 8.5 points after 8 weeks.

The PEP group showed an improvement of 2.0 points after 2 weeks and of 6.3 points after 8 weeks (Fig. 2b). Results of the PSQI show a mean difference compared to baseline of 1.5 points after 2 weeks and of 1.9 points after 8 weeks in the HTB group, and a deterioration of 0.1 points after 2 weeks and an improvement of 0.7 points after 8 weeks in the PEP group (Fig. 2c). Subgroup-analyses according to baseline depression severity in HAM-D score quartiles (median) revealed the greatest treatment effect in quartiles 3 and 4 (Table 5). There was no correlation between body temperature and outcome (data not shown).

**Table 5:** Subgroup-analyses according to HAM-D score quartiles

	HTB		PEP	
	Mean (SD)	n	Mean (SD)	n
**HAM-D ≥ 21**	−7.9 (3.7)	10	−3.4 (2.1)	5
**HAM-D ≥ 24**	−11.5 (2.7)	4	−3.0 (0.0)	2

### Global judgment of efficacy and tolerability

The global judgment by the patients showed no significant differences between the groups (Table 6), with good to moderate efficacy and tolerability after four interventions and at the end of treatment after 16 interventions.

**Table 6:** Global judgment of efficacy and tolerability

	HTB	PEP	p
Efficacy			
T1: 2 weeks	2.6 (1.2)	2.4 (1.4)	0.76
T2: 8 weeks	2.1 (1.0)	2.3 (1.1)	0.53
Tolerability			
T1: 2 weeks	2.1 (1.0)	1.6 (0.7)	0.16
T2: 8 weeks	1.8 (0.8)	1.7 (0.7)	0.66

### Adverse events

AE possibly related to the therapy were reported by 25 patients, of which 18/21 were assigned to the HTB group and 7/11 to the PEP group (Table 7). No SAE were reported by either group. There was no significant difference between the groups (*p*_2-tailed_ = 0.197). Typical AE in the HTB group were discomfort during the baths such as dizziness, fatigue, palpitations and thus mainly attributable to the cardiovascular system and indicating that the HTB were applied at a therapeutic limit. Additionally, patients reported transient effects as minor headache, itching, sweating, tightness, hunger worsening of depression and irritability. Typical AE in the exercise group were muscle soreness, pain in joints, the lower back or tendons; one patient reported cough and dizziness caused by breathing in cold air during exercise.

**Table 7:** Summary of adverse events

	HTB Group (***n*** = 21)		PEP Group (***n*** = 11)	
Adverse events	*n*	%	*n*	%
Dizziness during the intervention	13	62	1	9
Fatigue during the bath	6	29		
Palpitations during the bath	4	19		
Itching transient	4	19		
Heat/sweating transient	3	14		
Feeling of tightness, transient	2	10		
Headache, transient	2	10		
Hunger transient	1	5		
Insomnia in the night after the bath	1	5		
Pre-syncope after the bath	1	5		
Worsening of depressive symptoms in the night after the bath	1	5		
Irritability transient	1	5		
Muscle soreness transient			3	27
Joint pain transient			3	27
Sciatica transient			1	9
Tendon pain transient			1	9
Cough after sport			1	9

### Dropouts and compliance

AE that were not related to the treatment but which led to dropout included one patient in the HTB group who was hospitalized before the start of the treatment and six in the PEP group, three of which withdrew because of physical illness and three because of psychiatric hospitalization; three of these before the start of the treatment and the other three during the first 2 weeks of treatment.

Treatment related AE resulted in three dropouts in the PEP group (muscle pain); there were no treatment-related dropouts in the HTB group. Compliance of the PP population was good with a medium number of treatments of 13.3 (95% CI 11.6 to 14.9) in the HTB group and of 12.6 (95% CI 9.1 to 16.1) in the PEP group.

### Feasibility of HTB in a home-setting

After four sessions under supervision, the remaining 12 sessions were performed without supervision, either in a home-setting or at the thermal bath as before. The results show that HTB can be performed without supervision. However, only seven patients attempted to take the baths at home, and their main complaint was that it was difficult to reach the target water temperature of 40 °C; thus, they returned to the thermal bath.

## Discussion

To our knowledge, this is the first randomised controlled parallel group study to assess the efficacy of HTB compared with PEP in adults with depressive disorder. The main finding from this preliminary study is that HTB reduced the HAM-D score compared with PEP, but the effect was not significant in the PP-analysis. It should be emphasized that the onset of treatment response occurred within the first 2 weeks. Commonly used antidepressants, the standard treatment for depression, have a delayed onset of action. It requires weeks of treatment before the core symptoms of depression are ameliorated [9]. In SSRIs (selective serotonin reuptake inhibitors), i.e. fluoxetine, this discrepancy between antidepressant-induced acute neurochemical effects and clinical effectiveness adds up to 4 weeks in 75% of responders [8]. Consequently, HTB could offer a concomitant treatment option to close this gap. Exercise as a treatment for depression took 6 to 8 weeks to show an effect but this effect may be overlapped by the natural course of depression (Fig. 2). The between-group difference after 2 weeks, adjusted for baseline values, was 4.3 HAM-D points (*p* < 0.001 PP-analysis 2.7 points *p* = 0.07), which is a clinically relevant difference especially in comparison to an active non pharmacological therapy. As established by the National Institute for Clinical Excellence (NICE) the threshold for clinical relevance is a difference of 3 points on the HAM-D [39]. HAM-D total score was also lower at 8 weeks in the HTB group, but high attrition rates reduce confidence in this result. As in pharmacological studies, the magnitude of the difference in HAM-D scores between the HTB and the PEP group increased with increasing baseline depression severity. The PP analysis showed a trend towards better results in the PEP group at 8 weeks. This however, may be due to a selection bias as 13 patients dropped out in the PEP group. It also might be that hyperthermia has a rapid effect that is not further maximized by longer treatments.

The results of the HAM-D at 2 weeks are supported by the outcomes of the BDI-II, which also showed an improvement with an adjusted mean difference of 7.5 points (*p* = 0.002; PP-analysis 3.0 points *p* = 0.32) in favor of the HTB group, representing a decrease of 30% between the groups in favor of the HTB group after 2 weeks. However, after 8 weeks the HAM-D scores were similar in both groups.. According to the NICE-guidelines a difference of 3 points in the BDI-II, respectively a decrease of 17.5% compared to baseline is regarded as clinically relevant [39]. Whether the improvement in sleep in the HTB group after 2 weeks as seen in the PSQI is the cause or the consequence of the improvement in depression cannot be answered with this trial.

The patients’ overall global judgment of efficacy showed no difference between the groups. This is similar to the results from Button et al. [52], respectively the TREAD trial [53]. The global rating of change was “better” only with a corresponding mean change of − 18.9 in the BDI-II, whereas a mean change of − 6.4 was still regarded as “no” improvement.

There were fewer females in the HTB group (59%) than in the PEP group (87%); however, in a recent review gender did not modify the antidepressant effect of exercise [34]. In members of the PEP group, symptoms of depression had lingered for a longer period (8 years) than in those of the HBT group (4 years). Because longer duration of depression negatively affects treatment outcome the difference may have influenced the results [54].

Safety concerns existing prior to the study, especially regarding orthostatic dysregulation after HTB in the unsupervised part of the study, were not confirmed. We found some minor transient AE in both groups, but there were no SAE. Whether HTB is feasible in the home-setting cannot be answered conclusively, because only a few patients bathed there. Although not under investigation, the high attrition rate in the PEP group indicates that physical exercises are not suitable for everyone. Our finding of efficacy of the HTB intervention confirms the results of previous studies [12, 15, 21, 22].

### Strengths and limitations

The strengths of our study are the randomised, controlled design, the use of standardized baths, the good control of body temperature and the use of established patient as well as investigator-related questionnaires.

Several limitations should be discussed. First, the number of dropouts in the PEP group (56.5%) was far higher than in other trials investigating exercise in depression (18.1% dropouts). Higher baseline depressive symptoms are predicting a higher number of dropouts and dropout rates are likely to be higher in the outpatient, as in our, than in the inpatient setting [55]. In part, the dropouts in the PEP group are not related to the study (3 patients were hospitalized, 3 developed somatic disorders), but 3 dropped out because of muscle pain and some might have been not motivated. Exercise, therefore may not be suitable for all patients and HTB may for some be better accepted. Second, because of the small sample size, the study has limited power to detect clinically significant differences between the treatment conditions, especially in subgroup analyses. Third, the absence of blinding of treatment conditions, which is inherent and inevitable; due to unblinded outcome assessment a risk for performance bias exists. Patient preferences were tried to be kept on a minimum by balancing all information on the study interventions. Fourth, it is a well-known fact that the HAM-D total score has pitfalls; however, for better comparability with other studies, we did not use the GRID-HAM-D, e.g., with better reliability and validity [56, 57]. Fifth, the validity of paper diary records is limited. Concerns about compliance with paper diaries include poor adherence and retrospective or just-before-a-visit recording [58]. Sixth, there was no structured interview to come to the diagnosis depressive disorder. We relied on the diagnosis by a physician or psychotherapist treating the patients. Seventh, we did not investigate cold application after HTB or the mineral content in the water as potential modifiers of hyperthermia. This might be done in future studies.

### Generalizability

Although external validity may be restricted due to the population selected to participate in clinical studies, the population studied here can be regarded as representative of routine clinical practice, including patients with and without antidepressant medication [59]. Contraindications to HTB are still not well defined. Severe concomitant diseases, i.e. cardiovascular, or orthostatic dysregulation should be omitted, especially in the elderly.

## Conclusion

- HTB added to usual care may be a fast-acting, safe and easy accessible method leading to clinically relevant improvement in depression severity after just 2 weeks but effects may not be further increased with ongoing treatments.
- HTB performed at a thermal bath can be regarded as safe and feasible without external supervision, provided that the patients are informed concerning the potential risks, especially regarding orthostatic dysregulation.
- Patients can apply the method at their own responsibility.
- Exercise may not be feasible in some patients. HTB can also be practiced by patients with problems performing exercise training.

Replication of the results in a large, confirmative trial would clearly have important implications for public health in that the social and financial tolls of moderate to severe depression could be mitigated with a low-cost and easily accessible intervention with high acceptance by patients.

## Data Availability

The datasets analysed during the current study is available from the corresponding author on reasonable request.

## Abbreviations

BDI-II: Beck Depression Inventar II
CI: confidence interval
DRKS: German Clinical Trial Register
HAM-D: 17-item Hamilton Depression Rating Scale
HTB: hyperthermic baths
ITT: intention-to-treat
LOCF: last-observation-carried-forward
PEP: Physical Exercise Program
PP: per-protocol
PSQI: The Pittsburgh Sleep Quality Index
SD: standard deviation
WBH: whole body hyperthermia
